# Identification of a Novel NLRP12 Frameshift Mutation (Val730Glyfs^∗^41) by Whole-Exome Sequencing in Patients with Crohn's Disease

**DOI:** 10.1155/2024/5573272

**Published:** 2024-02-23

**Authors:** Jintong Chen, Yanni Huang, Huaning Chen, Qinyu Yang, Weiwei Zheng, Yanjun Lin, Mengli Xue, Chengdang Wang

**Affiliations:** ^1^Department of Gastroenterology, First Affiliated Hospital, Fujian Medical University, Fuzhou 350005, China; ^2^Fujian Clinical Research Center for Liver and Intestinal Diseases, Fuzhou 350005, China; ^3^Department of Gastroenterology, National Regional Medical Center, Binhai Campus of the First Affiliated Hospital, Fujian Medical University, Fuzhou 350005, China; ^4^Department of Rheumatology, First Affiliated Hospital, Fujian Medical University, Fuzhou 350005, China

## Abstract

*NLRP12* encodes the nucleotide-binding leucine-rich repeat-containing receptor 12 protein and has been linked to familial cold autoinflammatory syndrome 2 (FCAS2). Previous studies have reported that NLRP12 protein can dampen inflammatory responses in DSS-induced mice colitis. To date, only four alterations in the *NLRP12* gene have been associated with Crohn's disease (CD). Here, we reported a novel heterozygous *NLRP12* frameshift mutation (c.2188dupG, p.Val730Glyfs^∗^41) identified by whole-exome sequencing in the proband with CD. The Sanger sequencing confirmed that his sister and father also carried this *NLRP12* mutation, which cosegregated well with the CD phenotype. In silico analysis predicted this mutation to be disease-causing. Patients heterozygous for this mutation exhibited decreased NLRP12 protein levels in the peripheral blood and colon. Functional assays showed that mutant *NLRP12* plasmid-transfected HEK293T cells exhibited significantly lower *NLRP12* mRNA and protein levels than wild-type plasmid-transfected cells. The nonsense-mediated decay inhibitor NMDI14 significantly increased *NLRP12* mRNA and protein levels in mutant plasmid-transfected cells. Overall, our results demonstrated that this heterozygous *NLRP12* mutation (c.2188dupG) resulted in decreased NLRP12 expression, which might contribute to the mechanism underlying CD.

## 1. Introduction

Crohn's disease (CD, OMIM #266600) is a chronic and recurrent inflammatory bowel disease that can involve any part of the gastrointestinal tract. It is generally thought that CD is caused by an excessive immune response to intestinal microbiota and environmental factors in genetically susceptible individuals, although the definitive etiology remains unclear [1]. In recent years, genome-wide association studies (GWAS) and subsequent meta-analyses have identified over 140 genetic loci associated with CD susceptibility [2–5]. Among these loci, *NOD2* variants are the first-discovered and most well-established risk factor for CD [6]. The *NOD2* gene encodes an intracellular protein that contributes to the recognition and binding of intracellular bacterial peptidoglycan muramyl dipeptide (MDP), which subsequently recruits adaptor proteins to trigger a downstream signaling cascade involved in inflammation and the immune response [7]. Additionally, mutations in the genes regulating NOD2 downstream signaling (including *NPC* [8], *XIAP* [9], and *TRIM22* [10]) have been reported to be associated with increased susceptibility to CD. As a member of the nucleotide-binding domain leucine-rich repeat- (NLR-) containing family, NOD-like receptor 12 (NLRP12) plays an important role in pathogen recognition, host immunity, and inflammation [11–13]. Previous *nlrp12*-knockout mouse model studies have revealed that NLRP12 acts as a central component in maintaining intestinal homeostasis by inhibiting intestinal inflammation and tumor development [14, 15].

In this study, we identified a novel *NLRP12* heterozygous frameshift mutation in a Chinese family with CD using whole-exome sequencing (WES). We present detailed clinical data and further delineate the phenotype associated with CD. As the mutation cosegregated well with the CD phenotype, we further conducted several functional analyses of this mutation.

## 2. Materials and Methods

### 2.1. Patient Recruitment and Clinical Assessment

In this study, we investigated a Chinese family in which two members had been diagnosed with CD at the First Affiliated Hospital of Fujian Medical University. During colonoscopy screenings of their parents, it was found that the proband's father had also developed multiple aphthous ulcers in the terminal ileum, although he was asymptomatic. Informed written consent for this study was obtained from all family members, and the study protocol was approved by the Institutional Review Boards of First Affiliated Hospital of Fujian Medical University, China, in accordance with the Helsinki Declaration. Detailed clinical data, including demographic, clinical, endoscopic, and histological data, were collected from electronic medical records for each subject.

### 2.2. Whole-Exome Sequencing and Variant Screening

Peripheral blood was collected in ethylenediaminetetraacetic acid- (EDTA-) coated vacutainer tubes from each subject included in this study. Genomic DNA was extracted from blood using a DNA extraction kit (QIAamp DNA mini kit; Qiagen, Germany) according to the manufacturer's protocol, and then, DNA samples were fragmented by sonication and were processed with sequencing library preparation following the standard instructions by Illumina. After passing the sequencing quality controls with Q30 above 85% and Q20 above 90%, samples were sequenced with paired-end reads (PE150) using the NovaSeq 6000 platform (Illumina Inc., San Diego, CA, USA) to an average depth of at least 100×. The obtained FASTQ files were mapped to the human reference genome GRCh37 (hg19) using BWA v0.7.17. Several population databases, including the Exome Aggregation Consortium (ExAC) (http://exac.broadinstitute.org), Exome Sequencing Project (ESP) (http://evs.gs.washington.edu), and 1000 Genomes Project (1000 GP) (http://browser.1000genomes.org), were used to assess the allelic frequency of the potential causative variants in the general population. The two in silico tools MutationTaster (http://mutationtaster.org/MutationTaster/index.html) and Combined Annotation Dependent Depletion (CADD) (http://gs.washington.edu) were used to predict the pathogenic effect of the identified variant. PhyloP and PhastCons conservation scores were obtained from MutationTaster. Moreover, the identified mutation was classified and interpreted according to American College of Medical Genetics (ACMG) guidelines [16].

### 2.3. Immunofluorescence Staining of Bowel Biopsies

Formalin-fixed, paraffin-embedded, 5 *μ*m colonic biopsy sections of the proband and his family members were retrieved from the Pathology Department, First Affiliated Hospital of Fujian Medical University. Immunofluorescence staining was performed on sections as previously described [17]. Briefly, paraffin sections were immersed in xylene solution twice for deparaffinization and transferred to ethanol gradient solutions for rehydration. Afterward, the sections were placed in citrate antigen repair buffer and heated in a microwave oven for antigen retrieval. After cooling to room temperature, the sections were then incubated with 0.5% Triton X-100 in 1× PBS for membrane permeabilization. To inhibit nonspecific reactions, the sections were washed with PBS and blocked with 3% bovine serum albumin (BSA) in PBS for 30 min. Afterward, the sections were incubated at 4°C overnight with primary antibodies, including rabbit anti-NLRP12 (Affinity, China), rabbit anti-IL-1*β* (Bioss, Beijing, China), and mouse anti-NF-*κ*B p65 (Proteintech, USA). The sections were then rinsed and incubated with appropriate fluorescent-conjugated secondary antibodies at room temperature for 50 min. Secondary antibodies included Alexa Fluor 488- or CY3-conjugated goat anti-rabbit or goat anti-mouse antibodies (Servicebio, China). Cell nuclei were stained with 4′,6-diamidine-2′-phenylindole dihydrochloride (DAPI, Servicebio, China). Slides were visualized with a Nikon Eclipse C1 confocal microscope (Nikon, Japan) and scanned by a Pannoramic MIDI automated slide scanner (3DHistech, Hungary).

### 2.4. Plasmid Construction

The pcDNA3.1-NLRP12 wild-type (WT) expression plasmid was constructed by ligating the human full-length NLRP12 complementary DNA (cDNA) coding sequence and cloned into the expression vector pcDNA3.1-N-FLAG (Beyotime, China) with a 5′ Flag tag as described previously. The mutant pcDNA3.1-NLRP12-Val730Glyfs^∗^41 plasmid was generated by site-directed mutagenesis of the pcDNA3.1-NLRP12-WT plasmid according to the manufacturer's instructions. pcDNA3.1-N-FLAG was used as a negative control. The NF-*κ*B p65 expression plasmid was constructed as previously described [18]. All newly constructed plasmids were verified by DNA sequencing.

### 2.5. NF-*κ*B Luciferase Reporter Assay

Human embryonic kidney 293T (HEK293T) cells (1 × 10^5^) were transfected by using Lipofectamine 2000 (Invitrogen) with pNF-*κ*B-LUC luciferase reporter and the Renilla luciferase expression plasmid (internal control), together with NLRP12 WT or mutant expression plasmid or empty plasmid, respectively. The NF-*κ*B signaling pathway was induced by transfection of the p65 expression plasmid. After transfection for 24 h, cells were stimulated with or without TNF-*α* for another 16 h, and luciferase activities were detected in cell lysates by using a Dual Luciferase Reporter Gene Assay Kit (Beyotime, China) according to the manufacturer's instructions.

### 2.6. Real-Time qPCR

Total RNA was extracted from cells using NucleoZOL (Macherey-Nagel, Germany) and reverse transcribed to cDNA using the SweScript RT I First-Strand cDNA Synthesis Kit (Servicebio, China) according to the manufacturer's protocol. Quantitative RT–PCR (qRT-PCR) analysis was performed on an ABI 7500 system using the UltraSYBR Mixture (CWBIO, CW0957H) as previously described [19]. The PCR conditions were as follows: predenaturation at 95°C for 5 min and 40 cycles of denaturation at 95°C for 10 s, annealing at 60°C for 30 s, and extension at 72°C for 30 s, followed by a standard melt curve analysis.

The expression levels of target mRNA were normalized to an internal control (*β*-actin). The following primers were used for amplification: NLRP12: forward: 5′-TGCTTGTACGAGATCCAGGAG-3′ and reverse: 5′-CAGACAGAACGAGGAGACCAT-3′ and *β*-actin: forward: 5′-TGACGTGGACATCCGCAAAG-3′ and reverse: 5′-CTGGAAGGTGGACAGCGAGG-3′. The experiment was performed three independent times. The result was calculated using the 2-*ΔΔ*Ct method.

### 2.7. Western Blot Analysis

Total protein was extracted from cell lysates, and the protein concentrations were determined by a BCA kit (Solarbio, China). Protein lysates were denatured and separated in 8% SDS–PAGE gels and then transferred to polyvinylidene fluoride (PVDF) membranes (Millipore, USA). The membranes were blocked with 5% BSA in Tris-buffered saline with Tween 20 (TBST) for 1 h and were then incubated with primary antibodies against FLAG (Proteintech, USA), NLRP12 (Affinity, China), or *β*-actin (Abcam, UK) at 4°C overnight. Next, the membranes were rinsed with TBS-T three times and incubated with horseradish peroxidase- (HRP-) conjugated secondary antibody (Bioss, China) for 2 h at room temperature. Finally, the membranes were washed with TBS-T and visualized using an enhanced chemiluminescence kit (Thermo, USA), and the bands were scanned and analyzed with ImageJ analysis software (NIH, USA).

## 3. Results

### 3.1. Clinical Characteristics of Patients

#### 3.1.1. The Proband

**Figure 1:**
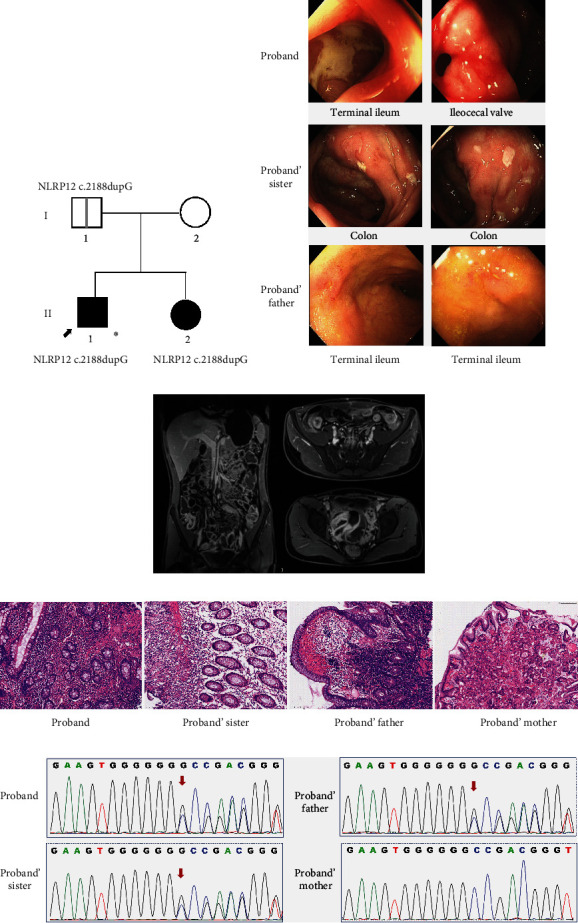
Identification of a novel *NLRP12* frameshift mutation in a Chinese family with Crohn's disease. (a) Pedigree of the studied family. The full black symbols indicate the affected individuals with clinical manifestations. The symbol with a gray vertical line inside denotes a presymptomatic status. The arrow indicates the proband, and the asterisk denotes the individuals in which WES was performed. (b) Colonoscopy images of the proband show a large and deep ulcer covered with white exudates in the terminal ileum and the stenosed ileocecal valve. Colonoscopy images of the proband's sister show multiple irregular active ulcers in the colon. Colonoscopy of the proband's father also revealed multiple aphthous ulcers in the terminal ileum. (c) Magnetic resonance enterography (MRE) of the proband shows segmental stenosis of the terminal ileum with thickening of the intestinal wall. (d) Biopsies from the terminal ileum of the colon of the patients exhibit prominent infiltration of inflammatory cells (mostly eosinophils and neutrophils), accompanied by a few epithelioid granulomas and focal ganglionitis in the mucosa and submucosa (hematoxylin and eosin (H&E), ×100). (e) DNA sequence electropherograms from the proband and his sister and father confirm a heterozygous G base pair duplication at position 2188 (c.2188 dupG) in the *NLRP12* gene. The red arrows show the c.2188 dupG mutation.

The proband was a 24-year-old Chinese man with a two-year history of chronic nonbloody diarrhea and intermittent abdominal pain. He presented to the emergency room with worsening symptoms. These symptoms prompted an abdominal computed tomography (CT) scan, which revealed bowel wall thickening in the terminal ileum with free gas in the peritoneal cavity. The findings were highly suggestive of intestinal perforation. He subsequently underwent an emergency exploratory laparotomy. During the laparotomy, approximately 200 ml of purulent ascitic fluid was found in the abdominal cavity. A thickened and edematous terminal ileum was noted in the lower right abdomen, and a perforation (2 mm in diameter) was present in the ileum approximately 30 cm proximal to the ileocecal region. After sufficient abdominal cleansing and drainage, the ileum perforation repair operation was performed. A colonoscopy 3-month postsurgery revealed stenosis of the ileocecal valve, which hampered insertion of the colonoscopy probe into the terminal ileum. A large, deep ulcer covered with white exudates in the terminal ileum was seen at the stenosed ileocecal valve orifice (Figure 1(b)), and the colon was normal. Biopsies obtained from the ileocecal valve revealed a mild chronic inflammatory infiltrate and crypt atrophy (Figure 1(d)). Magnetic resonance enterography (MRE) showed segmental stenosis of the terminal ileum with thickening of the intestinal wall (Figure 1(c)). Based on the above clinical data, he was subsequently diagnosed with CD and administered adalimumab subcutaneously every 2 weeks for 3 months without symptom improvement. Therefore, ustekinumab was then introduced. This treatment resulted in symptomatic relief and normalization of inflammatory indices.

#### 3.1.2. The Proband's Sister

The proband's sister was a 26-year-old woman who presented with recurrent periumbilical pain and loose normal-colored stool for the previous six months. She was subsequently diagnosed with CD with multiple irregular active ulcers in the colon, and a distorted and stenosed ileocecal valve was found by colonoscopy (Figure 1(b)). Histological findings showed prominent infiltration of inflammatory cells (mostly eosinophils and neutrophils), accompanied by a few epithelioid granulomas and focal ganglionitis in the mucosa and submucosa (Figure 1(d)). MRE revealed enhanced segmental wall thickening of the terminal ileum and colon and active perianal fistulas. She was subsequently treated with infliximab, and this treatment successfully resulted in clinical remission. Her ulcers were fully healed in subsequent colonoscopy evaluations.

#### 3.1.3. The Proband's Father

The proband's father was a 52-year-old Chinese man. He was asymptomatic, although multiple ulcers were detected in the terminal ileum during colonoscopy screening.

### 3.2. Genetic Analysis

To identify potential causal variants in this family, WES was carried out in peripheral blood from the proband. WES analysis revealed a novel heterozygous frameshift mutation (chr19:54310803 dupG hg19, NM_144687.3: c.2188dupG) in the *NLRP12* gene. The Sanger sequencing confirmed this heterozygous mutation and indicated that it was inherited from the proband's presymptomatic father and also carried by his affected sister (Figure 1(e)). This *NLRP12* c.2188 dupG variant was predicted to be disease-causing and to result in a frameshift at codon 730 that introduces a premature stop codon at codon 771, causing nonsense-mediated mRNA decay (NMD) as revealed by MutationTaster. The affected residues were checked for evolutionary conservation by multiple sequence alignment (PhyloP score 3.413 or PhastCons score 1). This variant (c.2188dupG (p.Val730Glyfs^∗^41)) was absent from the ExAC, ESP, 1000GP, and gnomAD population databases. According to ACMG guidelines [16], this variant is classified as pathogenic (PVS1, PM2, and PP1).

### 3.3. Protein Structure Prediction of the *NLRP12* Mutation

**Figure 2:**
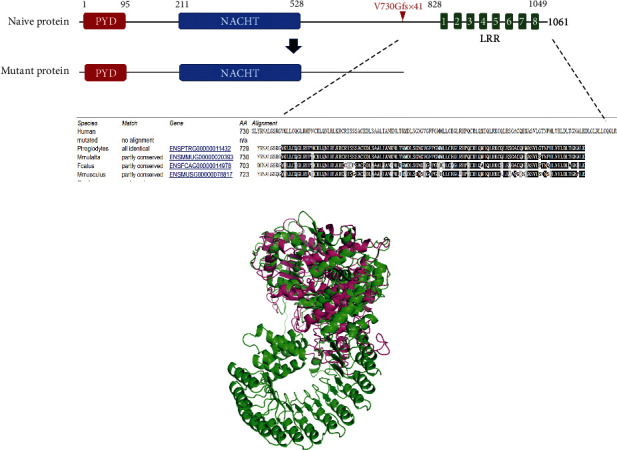
Prediction of the protein structure translated from mutated *NLRP12*. (a) Schematic diagram of the protein domains of human NLRP12 and the predicted mutant protein (p.Val730Glyfs^∗^41) due to the *NLRP12* c.2188dupG variant. Multiple alignment for the amino acid sequence of NLRP12 orthologs by MutationTaster. PYD: pyrin domain; NACHT: nucleotide-binding oligomerization domain; LRR: leucine-rich repeats. (b) The 3D structure model of the human native NLRP12 protein and mutant NLRP12 protein (p.Val730Glyfs^∗^41) were predicted using SWISS-MODEL online software. The green color represents the full-length NLRP12 protein. The purple one represents the truncated protein. It is evident that this mutation results in a protein product lacking the entire LRR domain in the C-terminus.

NLRP12 protein consists of three functional domains, the N-terminal pyrin domain (PYD), the central NACHT domain, and the C-terminal leucine-rich repeat (LRR) region (Figure 2(a)). The p.Val730Glyfs^∗^41 mutation, located between NACHT and LRRs, would generate a truncated NLRP12 protein lacking the whole LRR region, which is highly evolutionarily conserved (Figure 2(a)). The 3D protein structures of human native NLRP12 protein and its truncated form were predicted by the online SWISS-MODEL homology modeling software (http://swissmodel.expasy.org). This p.Val730Glyfs^∗^41 mutation results in a protein product lacking the entire LRR domain in the C-terminus (Figure 2(b)).

### 3.4. Immunofluorescence Staining of Bowel Biopsies

**Figure 3:**
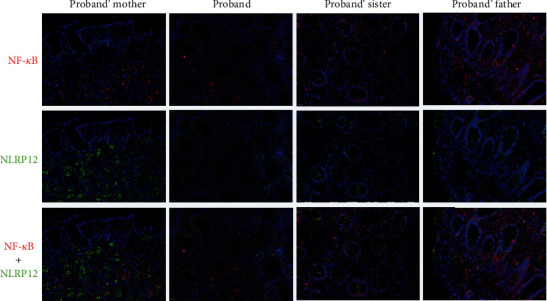
Dual immunofluorescence staining for NLRP12 protein and NF-*κ*B protein in the bowel biopsy tissues of these family members. DAPI (blue) was used as a counterstain for cell nuclei. In tissue sections from the proband and his sister and father, who carried the heterozygous p.Val730Glyfs^∗^41 frameshift mutation, the NLRP12 signal (green) was significantly weaker than that of his mother (heathy control). The NF-*κ*B p65 signal (red) was widely and highly detected in both the nucleus and cytoplasm in the epithelial and interstitial cells of the affected patients. NF-*κ*B p65 was detected only in the cytoplasm of the proband's mother.

Immunofluorescence staining of bowel biopsies was performed to analyze NLRP12, IL-1*β*, and NF-*κ*B expression in the bowel of these family members (Figure 3). In sections taken from the proband's mother, who did not carry the mutation, NLRP12 was strongly expressed in the intestinal mucosa. Notably, NLRP12 was expressed predominantly in the cytoplasm, consistent with data from the Human Protein Atlas. In the proband and in his sister, who carried the heterozygous p.Val730Glyfs^∗^41 frameshift mutation, NLRP12 protein expression was markedly decreased in epithelial and interstitial cells. We then tested IL-1*β* expression levels in the colon tissues of the patients. Immunofluorescence staining showed that IL-1*β* was highly and ubiquitously expressed in patient lymphocytes and intestinal tissues compared with those of the proband's mother (Supplementary Figure S1). Previous studies have reported that the NLRP12 protein acts as a repressor of the NF-*κ*B signaling pathway [20–23]. NF-*κ*B p65 was widely and highly expressed in both the nucleus and the cytoplasm in the epithelial and interstitial cells of the affected patients. However, in colon tissues from the proband's mother, NF-*κ*B p65 was detected only in the cytoplasm, suggesting reduced NF-*κ*B p65 nuclear translocation. These results indicated that NLRP12 protein expression decreased, while NF-*κ*B activation and IL-1*β* expression increased in affected individuals with the heterozygous p.Val730Glyfs^∗^41 mutation.

### 3.5. Functional Validation of NLRP12 Mutation

**Figure 4:**
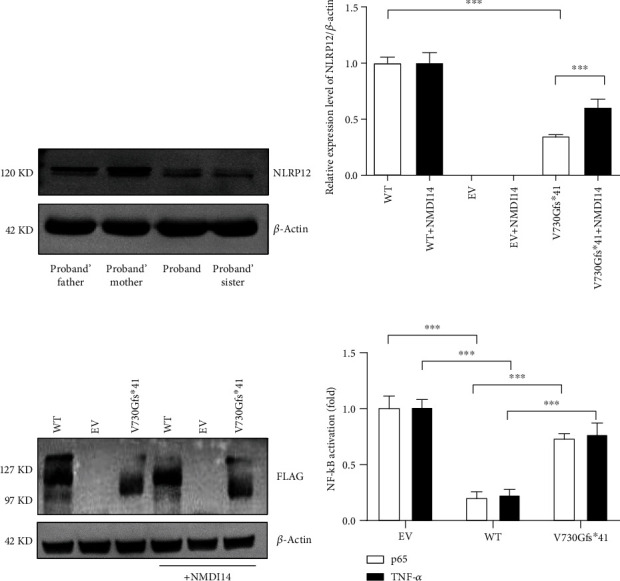
Functional consequences of the identified *NLRP12* mutation. (a) The WB results showed that NLRP12 protein levels in the peripheral blood of the proband and his father and sister were significantly lower than those of the heathy control (his mother). (b) The mRNA level of *NLRP12* transcribed from cells transfected with the mutant V730Gfs^∗^41 plasmid was significantly lower than that from cells transfected with the WT plasmid. NMDI14 significantly increased *NLRP12* mRNA transcribed from the V730Gfs^∗^41 plasmid but made no difference in mRNA levels transcribed from the WT plasmid. (c) The WB results showed that the expression of the mutant FLAG-NLRP12-V730Gfs^∗^41 protein (~97 kDa) was significantly lower than that of the NLRP12-WT protein (~127 kDa). Mutant protein levels significantly increased after treatment with NMDI14, but the treatment caused no difference in WT protein levels. (d) Effects of NLRP12-WT and NLRP12-mutant proteins on NF-*κ*B activity. The NF-*κ*B transcriptional activity induced by p65 was significantly suppressed by overexpression of NLRP12-WT protein but was almost rescued by NLRP12 V730Gfs^∗^41 protein. Similarly, the NLRP12-WT protein also inhibited TNF-*α*-induced NF-*κ*B activation, and this inhibition was dramatically abrogated by the NLRP12-mutant protein. WT: wild type; EV: empty vector; V730Gfs^∗^41: FLAG-NLRP12-V730Gfs^∗^41; WB: Western blotting; mRNA: messenger RNA. ^∗∗∗^ represents a *p*-value less than 0.01.

Western blotting was first performed to assess the expression of NLRP12 protein in the peripheral blood of these family members. Lower protein expression of NLRP12 was found in patients with heterozygous p.Val730Glyfs^∗^41 mutation compared to heathy control (Figure 4(a)).

We next constructed WT and mutant NLRP12 expression plasmids fused with a 5′-FLAG tag to investigate the potential functional consequences of the identified variant in HEK293T cells. The level of mRNA transcribed from the pcDNA3.1-NLRP12-Val730Glyfs^∗^41 plasmid was significantly lower than that from the WT plasmid, comprising 34% of the amount of mRNA transcribed from the WT plasmid (Figure 4(b)). An anti-FLAG antibody was used to detect the protein expression translated from the WT and mutant NLRP12 plasmids in Western blotting. Western blotting revealed that the truncated protein (~97 kDa) was detected in cells transfected with the mutant plasmid and was expressed at a level lower than that translated from the WT plasmid (Figure 4(c)). Furthermore, the aforementioned experiments were conducted iteratively on the human HT-29 intestinal epithelial cell lines, yielding consistent outcomes (Supplementary Figure S2). These results indicated that the NLRP12 p.Val730Glyfs^∗^41 mutation altered its gene and protein expression, thus resulting in loss of NLRP12 function.

### 3.6. Degradation of Mutant mRNAs by NMD

To investigate whether the reduction in the abundance of *NLRP12* mRNA harboring the c2188duG mutation is due to NMD, HEK293T cells were transfected with WT and mutant plasmids and then treated with NMDI14 for 12 h. NMDI14 is a small molecule inhibitor that is able to stabilize mutant mRNAs by disrupting the interaction between UPF1 and downstream NMD factors that modulate nonsense-mediated mRNA decay [24]. Treatment of HEK293T cells with NMDI14 significantly increased the level of FLAG-NLRP12 mRNA transcribed from the mutant plasmid but had no effect on the FLAG-NLRP12 mRNA level transcribed from the WT plasmid (Figure 4(b)). A similar pattern of FLAG-NLRP12 protein was detected by Western blotting (Figure 4(c)). In addition, intervention with NMDI14 also enhanced the protein expression level of mutant plasmids in HT-29 cells (Supplementary Figure S2). These results suggested that NMDI14 inhibited the degradation of mutant *NLRP12* mRNA.

### 3.7. NF-*κ*B Luciferase Reporter Assay

Dual luciferase reporter assays were next conducted to determine the role of *NLRP12* mutation on inhibition of NF-*κ*B activity. As presented in Figure 4(e), p65-induced NF-*κ*B transcriptional activity was significantly suppressed by overexpression of NLRP12-WT protein but was almost rescued by the frameshift mutation of *NLRP12* (p.Val730Glyfs^∗^41). Similarly, *NLRP12* mutation also dramatically abrogated the inhibitory effect of WT NLRP12 on NF-*κ*B activity after stimulation with TNF-*α*.

## 4. Discussion

In the present study, we identified a novel heterozygous *NLRP12* frameshift mutation (Val730Glyfs^∗^41) in patients with CD by WES. The *NLRP12* mutation was present in 3 members of this family, two of whom were diagnosed with CD due to typical clinical manifestations, endoscopic findings, and radiological and histological features. The proband's father also carried the variant and had asymptomatic ulcers in the terminal ileum.

The novel frameshift mutation c.2188dupG (p.Val730Glyfs^∗^41) in *NLRP12* resulted in a premature termination codon that is predicted to generate a truncated protein of NLRP12 lacking the entire LRR domain in the C-terminus or render the aberrant mRNA vulnerable to nonsense-mediated decay (NMD), leading to the loss of function of the NLRP12 protein. Herein, we found that patients carrying heterozygous *NLRP12* frameshift mutations expressed lower NLRP12 protein levels than heathy control in the peripheral blood and colon. The p.Val730Glyfs^∗^41 mutation significantly reduced the mRNA and protein levels of NLRP12 in transfected HEK293T cells, and treatment of cells with NMDI14 significantly increased *NLRP12* mRNA and protein levels. These data support that mutant *NLRP12* mRNA expression was subject to regulation by NMD. Based on the dominant inheritance pattern in this family and the decreased NLRP12 protein expression in affected patients, we speculate that this heterozygous loss-of-function mutation resulted in *NLRP12* haploinsufficiency, which might contribute to the mechanism underlying CD.

As a member of the NLRP (NOD, LRR, and PYD containing) subfamily, NLRP12 (previously known as NALP12, RNO2, PYPAF7, and Monarch1) is predominantly expressed in myeloid cells, including monocytes/macrophages, neutrophils, dendritic cells, and eosinophils [25]. To date, NLRP12 has been reported to have dual functions in innate immunity and inflammation. Previous studies have suggested that NLRP12 can form a multiprotein complex called the NLRP12 inflammasome, which is responsible for caspase-1 activation and the subsequent release of mature proinflammatory interleukin- (IL-) 1*β* and IL-18 in response to *Yersinia* and *Plasmodium chabaudi* [11, 12]. In contrast, increasing evidence indicates that NLRP12 exerts a dominant-negative regulatory effect on inflammatory processes by inhibiting the canonical and noncanonical NF-*κ*B and extracellular signal-regulated kinase (ERK) activation pathways [20–23]. NLRP12 has been implicated in the regulation of intestinal inflammation in mice. Previous studies have revealed that *nlrp12*-deficient mice are significantly more susceptible to dextran sodium sulfate- (DSS-) induced colitis, which was attributed to increased canonical and noncanonical NF-*κ*B activation and enhanced expression of inflammatory cytokines and chemokines [14, 15]. Additionally, another study conducted by Ting et al. demonstrated that *nlrp12* deficiency in mice increased basal colonic inflammation and dysbiosis, as evidenced by loss of beneficial *Bacteroidales*, *Clostridiales*, and *Lachnospiraceae* and increased abundance of colitogenic *Erysipelotrichaceae*. Overall, these data suggest that NLRP12 functions as a negative regulator of colon inflammation by maintaining colonic microbial diversity and promoting beneficial commensal bacterial growth [26]. In this study, we showed that *NLRP12* p.Val730Glyfs^∗^41 mutation abolished its NF-*κ*B suppressive activity, which may subsequently lead to intestinal hyperinflammation in CD.

In humans, mutations in *NLRP12* have been linked to familial cold autoinflammatory syndrome 2 (FCAS2, OMIM #611762), which is characterized by recurrent, cold-induced episodes of fever associated with various systemic symptoms, including urticaria, headache, myalgia, arthralgia, and joint swelling [27–33]. In addition, patients with *NLRP12* mutations can also present with abdominal pain, vomiting, and buccal aphthous ulcers together with cold-induced fever [28, 34]. Another study suggested that *NLRP12* mutations might account for a small proportion of common variable immunodeficiency in patients with severe autoinflammatory complications [35]. To date, only four *NLRP12* variants have been reported in five patients with CD (Supplementary Table S1). Tal et al. [36] described a patient with severe herpes simplex virus (HSV) esophagitis and CD. WES analysis revealed that he had two coinciding genetic mutations in toll-like receptor 3 (*TLR3*) and *NLRP12*. Although the exact mechanistic links between these two genes and his clinical phenotypes could not be determined, the heterozygous frameshift mutation (c.1113_1116delGGAA) in the *NLRP12* gene may partially account for his CD-like clinical manifestations. More recently, Jyonouchi and Geng [37] also presented the case of a female patient with an *NLRP12* mutation (C.1054C>T) in addition to a variant of interferon regulatory factor 2 binding protein 2 (*IRF2BP2*). She was initially diagnosed with pediatric acute-onset neuropsychiatric syndrome (PANS), with favorable responses to pulsed oral corticosteroid treatment and subsequent intravenous immunoglobulin administration due to her immunoglobulin-G1 deficiency. However, she subsequently developed CD and poor responses to TNF-*α* blocker treatment. Her illness was finally controlled by treatment with ustekinumab. Unfortunately, the authors of that study did not provide genetic pedigree information or conduct a functional analysis of the *NLRP12* mutation. Unlike previous reports, the three family members carrying the NLRP12 heterozygous mutation in our study manifested neither overt evidence of immunodeficiency nor typical clinical manifestations of familial cold autoinflammatory syndrome, such as recurrent periodic fever, urticaria, myalgia, and arthralgia. Additionally, the three affected members exhibited varying gastrointestinal symptoms. Notably, despite multiple ulcers in his intestine, the father remained asymptomatic. This discrepancy between genotype and clinical phenotype could be attributed to various factors, including dietary, environmental, and epigenetic influences, underscoring the complexity of the NLRP12 gene mutation.

Furthermore, the proband and his sister in our study received treatment with ustekinumab and infliximab, respectively. Despite targeting different molecular targets, both drugs successfully induced clinical remission in the patients. This could be attributed to the intersecting downstream signaling pathways influenced by the molecular consequences of the shared mutations they carry in distinct ways.

Identifying pathogenic gene mutations and gaining a deeper understanding of the molecular pathway alterations caused by these mutations may contribute to the development of innovative and personalized therapeutic strategies. Currently, there is no targeted therapy available for NLRP12 deficiency. Allogeneic hematopoietic stem cell transplantation (HSCT) has been successfully employed in some very early onset-IBD (VEO-IBD) patients, as exemplified by IL-10R deficiency [38]. Routine WES screening in VEO-IBD patients can identify specific IBD patient subtypes, providing opportunities for tailored individualized treatments [39].

Our study also presents several potential limitations. Firstly, there is a need for further investigation into the precise molecular mechanism that underlies the pathogenesis of Crohn's disease caused by the NLRP12 mutation (Val730Glyfs^∗^41). Secondly, conducting animal experiments using gene-targeting mutant mice would strengthen the credibility of our findings.

## 5. Conclusions

In conclusion, we first identified a novel heterozygous *NLRP12* mutation (p.Val730Glyfs^∗^41) in Chinese patients with CD. Functional analysis revealed that this heterozygous mutation leads to a decrease in *NLRP12* expression, possibly due to degradation via NMD, and it ultimately causes haploinsufficient loss of function of the inhibition of NF-*κ*B activity. This study expands the clinical phenotypic spectrum of *NLRP12* deficiency. Thus, *NLRP12* gene mutation should be considered in the diagnostic work-up in patients with suspected monogenic CD, even in those without the typical systemic inflammatory manifestations of FACS. Further studies are warranted to better clarify the role of NLRP12 in CD pathogenesis and to determine potential effective therapeutic strategies.

## Data Availability

The data that support the findings of this study are available from the corresponding author Chengdang Wang (wangcdhl@fjmu.edu.cn) upon reasonable request.

## Supplementary Materials

Supplementary materials Supplementary 1. Table S1: summary of reports of Crohn's disease associated with NLRP12-mutations. Supplementary 2. Figure S1: immunofluorescence staining for IL-1*β* protein in the bowel biopsy tissues of these family members. Supplementary 3. Figure S2: Western blotting analysis of protein expression in HT29 cells.
