# Supplementary material for: Identification of a Novel NLRP12 Frameshift Mutation (Val730Glyfs∗41) by Whole-Exome Sequencing in Patients with Crohn's Disease
Source: Hum Mutat. 2024 Feb 23;2024:5573272. doi: 10.1155/2024/5573272 (PMC11918926; doi:10.1155/2024/5573272)
Supplement: Supplementary materials — Supplementary 1. Table S1: summary of reports of Crohn's disease associated with NLRP12-mutations. Supplementary 2. Figure S1: immunofluorescence staining for IL-1β protein in the bowel biopsy tissues of these family members. Supplementary 3. Figure S2: Western blotting analysis of protein expression in HT29 cells. [file 5573272.f1.docx]

Table S1. Summary of reports of Crohn’ disease associated with *NLRP12-*mutations.

| P | **S** | **Nucleotide change/Amino acid change** | **Zygo** | **Variant type** | **Other concomitant gene variants** | **Origin** | **symptoms** | **Comorbidities** | **Treatment** | **References** |
| --- | --- | --- | --- | --- | --- | --- | --- | --- | --- | --- |
| P1 | M | c.910C > T, p.H304Y | HET | Ms | **-** | Russian | PF, AP, CV | Food allergy | TNFα inhibitors | (Kostik et al. 2018) |
| P2 | F | c.910C > T, p.H304Y;  c.1206C>G, p.F402L | HET | Ms | **-** | Russian | PF | CVID, AIHA, pancytopenia, S, ILD, IS | IVIG, CS | (Kostik et al. 2018) |
| P3 | M | c.1206C>G, p.F402L | HOM | Ms | *NOD2*: c.3019dupC, p.L1007fs | Russian | PF, AP, S, FT | S | TNFα inhibitors | (Kostik et al. 2018) |
| P4 | F | c.1113_1116delGGAA, p.L371Afs*20 | HET | Fs | *TLR3*: c.1234C>T, p.L412P | non-consanguineous Jewish | AP, ID, CP | HSV-1 esophagitis,  Epilepsy, Hypertension | Acyclovir, CS, Vedolizumab | (Tal et al. 2020) |
| P5 | **F** | C.1054C>T, p.A352C | HET | Ms | *IRF2BP2*：c.1180A>C, p.T394P | non-Hispanic Caucasian | AP, H, A, ID | CVID, IS | CS, IVIG, TNFα inhibitors, Anakinra, Ustekinumab | (Jyonouchi and Geng 2022) |
| P6 | M | c.2188duG, p.V730Gfs*41 | HET | Fs | - | Chinese Han | AP, ID | - | Ustekinumab | In this study |
| P7 | F | c.2188duG, p.V730Gfs*41 | HET | Fs | - | Chinese Han | AP，ID | - | TNFα inhibitor | In this study |
| P8 | M | c.2188duG, p.V730Gfs*41 | HET | Fs | - | Chinese Han | - | - | - | In this study |

P, Patient; S, sex (M, male; F, female); Ms, missense; Fs, frameshift; Zygo, zygosity; HET, heterozygous; HOM, homozygous; PF, periodic fever; AP, abdominal pain; CV, сyclic vomiting; S, splenomegaly; FT, failure to thrive; ID, intermittent diarrhea; CP, chest pain; H, headache; A, arthralgia; CVID, common variable immunodeficiency; AIHA, autoimmune hemolytic anemia; ILD, interstitial lung disease; IS infection susceptibility; HSV-1, herps simplex virus-1; IVIG, intravenous immunoglobulin; CS, corticosteroids


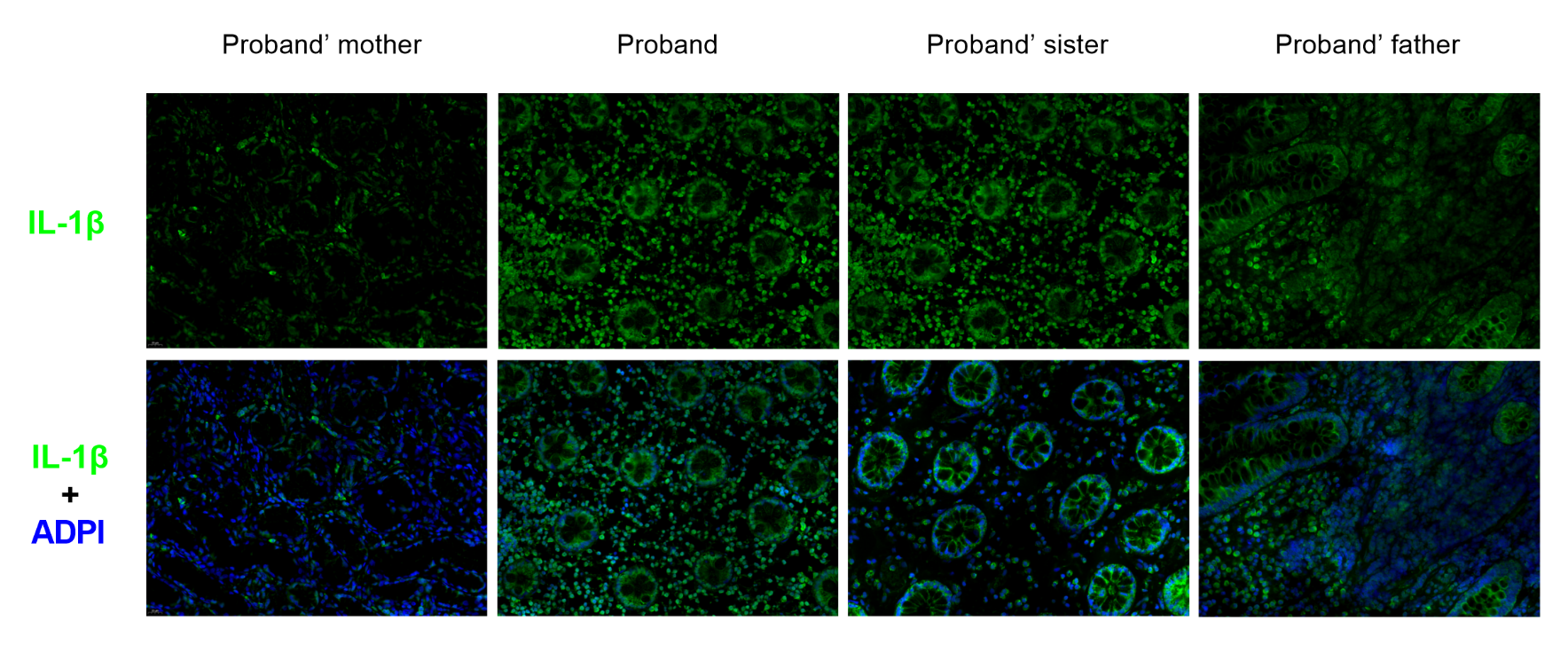


Figure S1：Immunofluorescence staining for IL-1β protein in the bowel biopsy tissues of these family members. DAPI (blue) was used as a counterstain for cell nuclei. In tissue sections from the proband and his sister and father, who carried the heterozygous p.Val730Glyfs*41 frameshift mutation, the NLRP12 signal (green) was significantly stronger than that of his mother (heathy control).


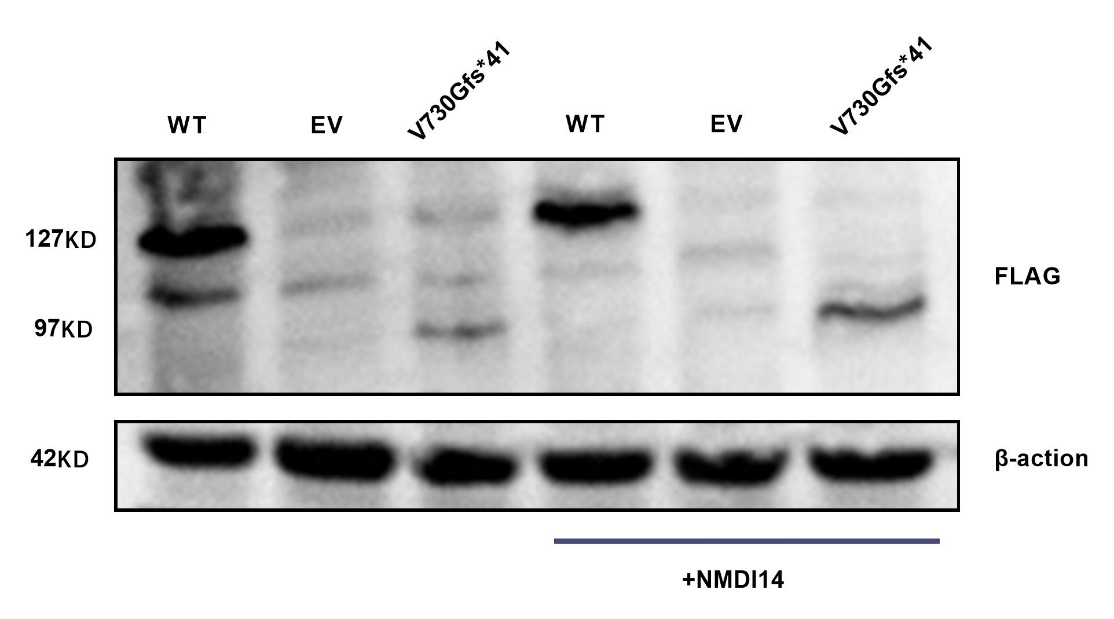


Figure S2: Western blotting (WB) analysis of protein expression in HT29 cells. The results of the WB showed a significant decrease in protein expression of the transfected FLAG-NLRP12-V730Gfs*41 mutant plasmid compared to the transfected wild-type NLRP12 plasmid. Additionally, when treated with NMDI14, the mutant plasmid-transfected cells exhibited a significant increase in protein expression levels. WT: wild type; EV: Empty vector; V730Gfs*41: FLAG-NLRP12-V730Gfs*41; WB: Western blotting.

**References**

Jyonouchi H, Geng L (2022) Whole-exome sequencing in a subject with fluctuating neuropsychiatric symptoms, immunoglobulin G1 deficiency, and subsequent development of Crohn’s disease: a case report. Journal of Medical Case Reports 16: 187. doi: 10.1186/s13256-022-03404-9

Kostik MM, Suspitsin EN, Guseva MN, Levina AS, Kazantseva AY, Sokolenko AP, Imyanitov EN (2018) Multigene sequencing reveals heterogeneity of NLRP12-related autoinflammatory disorders. Rheumatol Int 38: 887-893. doi: 10.1007/s00296-018-4002-8

Tal Y, Ribak Y, Khalaila A, Shamriz O, Marcus N, Zinger A, Meiner V, Schuster R, Lewis EC, Nahum A (2020) Toll-like receptor 3 (TLR3) variant and NLRP12 mutation confer susceptibility to a complex clinical presentation. Clin Immunol 212: 108249. doi: 10.1016/j.clim.2019.108249
